# The Frontier of Penile Implants in Phalloplasty: Is the ZSI 475 FTM what we have been waiting for?

**DOI:** 10.1038/s41443-020-00396-2

**Published:** 2021-01-22

**Authors:** Mirko Preto, Gideon Blecher, Massimiliano Timpano, Paolo Gontero, Marco Falcone

**Affiliations:** 1 Urology Department – A.O.U. “Città della Salute e della Scienza” – Molinette Hospital, University of Turin Italy, Corso Bramante 88, 10126 Turin, Italy; 2 Adjunct Lecturer, Department of Surgery. Monash University, Melbourne (Australia) Wellington Rd, Clayton, VIC 3800 Australia; 3 Urology Consultant. Department of Urology, The Alfred Hospital. Melbourne, (Australia), 55 Commercial Rd, Melbourne, VIC 3004 Australia; 4 Spinal Cord Unit – Department of Neurourology - A.O.U. “Città della Salute e della Scienza” – Molinette Hospital, University of Turin, Via Zuretti 24, 10126 Turin, Italy

**Keywords:** Quality of life, Surgery

Penile prosthesis (PP) implantation represent ts the final stage of neo-phallus creation, and is arguably the most exciting step for a transgender patient. It transforms an otherwise aesthetic organ, which may or may not provide voiding capabilities, into a functional sexual organ. This aspect remains an essential component for some trans-males undergoing gender genital affirming surgery (GGAS), defining their masculinity and supporting their quality of life [1, 2].

Whilst there have been advances in phalloplasty techniques, as well as developments in the implant technologies, there unfortunately remains significant complications which must be stomached by both patients and health care providers. If these issues were to be resolved, say by the ideal PP, one would assume that both patients and surgeons would more readily access phalloplasty as part of gender dysphoria management. Unfortunately however, the perfect prosthesis, particularly in phalloplasty, eludes us. Ideally it would be completely inert and remain free from infection. It would also provide perfect rigidity and stability when erect and be easily incorporated into the pelvis. Continuing our dream of a problem free implant, it would remain permanently free from mechanical damage and would never find its way eroding through local tissues. So what is the reality for trans-males undergoing PP implantation in a phalloplasty?

In terms of PP, both malleable and inflatable devices have been implanted. Hydraulic implants are often utilised due to the more natural appearance in the flaccid state, as well as because of the reduced apical pressure on the phallic tissues, thus minimising the risk of distal erosion [3–5]. It is not only erosion that poses a problem: it seems inequitable that cis-males whom require PP, whilst experiencing potential complications, are much less exposed to such problems than trans-gender males. The incidence of postoperative complications are dramatically higher in phalloplasty, compared to a native phallus [6–9].

The main surgical complications remain implant infection, erosion or malposition. Patient reported outcomes (PROs) of interest are varied but many would include sensation, orgasmic function, ability to engage in penetrative intercourse, as well as overall satisfaction, as key measures. Whilst there is extensive literature regarding PP in cis-males, the research documenting outcomes in phalloplasty is much more limited.

Six [3–6, 10–12] major studies (implant *n* > 40) have been published on this topic, most of them being relatively small volume (implant *n* < 247, mean patient *n* = 97), retrospective, with high heterogeneity in terms of implant models, techniques and outcome measures [3–6, 10–12] (Table 1).

**Table 1:** Larger series of penile prosthesis in male-transgender phalloplasty.

Author	Year	Patients (number)	Follow up (months)	Implants (number)	Model of penile implant (number, %)	Overall Revision rate (%)	Infection (%)	Mechanical failure (%)	Dysfunction/malposition (%)
Falcone [3]	2016	247	20	328	AMS700CX (226, 68.9%) AMS 700 CXM/R (31, 9.4%) Coloplast Titan (58, 17,7%) Ambicor (13, 3,9%)	43,3	8,5	15,4	19,4
Hoebeke [4]	2009	129	30,2	185	Dynaflex (9, 6.9%) AMS700CX/CXM (50, 38.7%) AMS700CX Inhibizone (17, 13.3%) Ambicor (47, 36.5%) Coloplast Titan (6, 4.6%)	41,1	11,9	22	- Erosion 8,1 - Malposition 14,6 TOT 22,7
Van der Sluis [5]	2019	32	55	45	Dynaflex (13, %) Ambicor (3, %) Coloplast Genesis (14, %) AMS Spectra (2, %)	44	17,8	11,1	- Erosion 6,7 - Malposition 4,4 TOT 11,1
Neuville [6]	2016	69	48	95	AMS Ambicor (90, 94.8%) AMS 700 CXR/CX/600 (5, 5.2%)	37,7	9,6 (early and late infection)	10,5	- Erosion 4,2 - Malposition 12,6 TOT 16,8
Djordjevic [11]	2019	129	43	61	Coloplast Genesis 39 AMS 700LGX 22	6,6	3,3	5,1	NA
Leriche [13]	2008	38	110	49	Ambicor AMS 600 AMS 700 S	37	29 (mechanical failure included)	29 (infection included)	NA
Neuville [14]	2019	20	9	21	ZSI 475 FtM	18,9	4,7	9,5	4,7
Verla [15]	2020	46	12	46	ZSI 475 FtM	20	11	4,3	- Erosion 2,2 - Malposition 4,3 TOT 6,5

Survival rates for PP in phalloplasty have remained significantly lower than in non-phalloplasty cohorts, with 5 year PP survival ranging from 75 to 78% [3, 5]. Infection thus remains a common reason for explant, occurring in about 8.5–17.8% of cases [3–6, 11, 12]. Contemporary comparative infection rates in non-phalloplasty groups, remain much lower and vary from 1.1 to 1.7% [6].

Mechanical failure, cylinder malposition and erosion rates reported in GGAS are unfortunately significantly higher than those reported in genetic males. Mechanical failure develops in 5 to 22% of cases [3–6, 11, 12], malpositioning ranges from 4.4-19.4% [3–6] and erosion occurs in 4.2-8.1% [4–6].

These higher incidences of complications results in more frequent PP revision surgery, which has been described ranging from 37 to 44% (mean follow up 45 months). Only Djordjevic et al. [11] in their series reported a significant lower revision rate, of 6.6% (mean follow up 43 months).

Regarding functional outcomes, data are even more sparse; few studies have analyzed the satisfaction rates and PROs. In the two studies which reported device cycling, all patients were able to cycle the PP [3, 11]. Despite significant complications as described above, overall satisfaction rates reached 88% [3], remarkably comparable to non-phalloplasty males undergoing PP. Phalloplasty patients have been able to engage in penetrative intercourse in 51–77% of cases [3, 12].

## Whilst complications are generally poorer, the operation has its own specific hurdles

The anatomical lack of corpora cavernosa is one of the major differences when implanting prostheses in trans-males. Indeed, the lack of the tunica albuginea, which normally forms a protecting envelope around the cylinders, may affect their durability. Until recently, surgeons had to design their own makeshift solutions to overcome the absence of natural housing for the cylinders.

To avoid erosion, Mukherjee described an incorporated pouch, into which a removable prosthesis can be placed when required [13]. More commonly nowadays, surgeons created a makeshift prosthetic Dacron sock, to anchor the proximal cylinder to the pubic bone. Unfortunately, Dacron may be in part to blame for increasing the risk of infection and PP mechanical failure [3, 4].

Looking back at their clinical experience, most of the reconstructive surgeons complained of the absence of a specifically designed PP for phalloplasty over years [3, 12]. Common thought was that having a specifically designed device for transgender patients with specific materials and technical features could have reduced the incidence of complications.

**Fig. 1:**
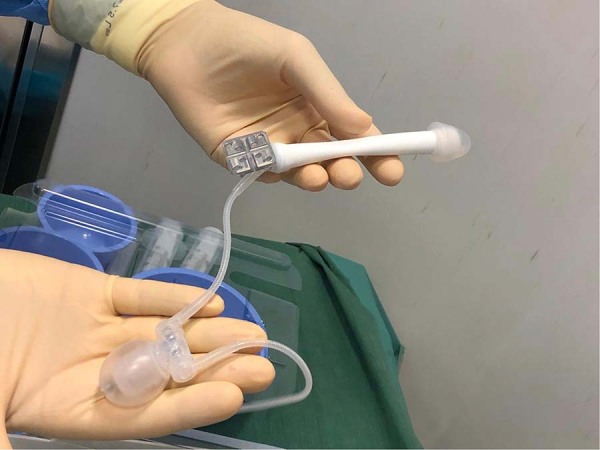
ZSI 475 FTM penile implant: the aspect of the device.

**Fig. 2:**
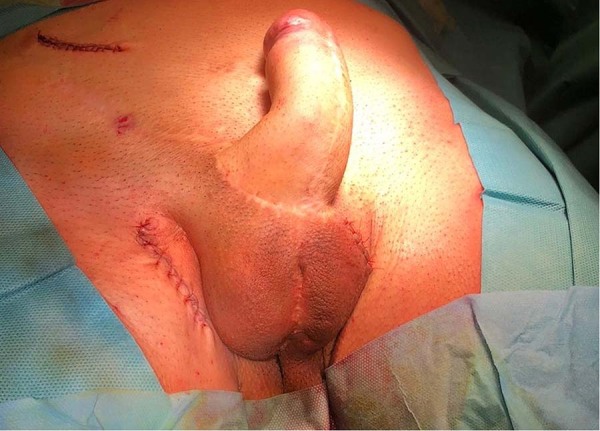
The final aspect of the neophallus after ZSI 475 FTM implantation.

Only recently in 2016, was a phalloplasty specific implant ZSI-475 FTM (Zephyr Surgical Implants, Geneva, Switzerland) designed to address some of these issues [14]. It is composed by a single inflatable cylinder with the diameter of 21 mm protected by a distal glans-shaped stopper of 25 mm. Moreover, it provides an incorporated anchorage plate, in the proximal part of the cylinder, composed of stainless steel and silicone to be sealed with four non-absorbable stitches to the pubic periosteum. These two features removed the necessity for synthetic socks and caps. Finally, the pump is testicle-shaped to improve aesthetical appearance of the scrotum (Figs. 1 and 2). It was also hoped to reduce the intraoperative time as well as the risk of infection, mechanical failure and erosion since no foreign materials are in contact with the PP.

The data on the ZSI 475 FTM rests upon our own outcomes as well as two other published papers [14–16]. The first report of the ZSI implant in 2016 (*n* = 20), reported 80% implant survival at a mean of 9 months. Infection (4.7%), mechanical failure (9.5%) and malposition (4.7%) were causes for explant [14].

A major limitation when evaluating the efficacy of PP implantation in transgender men is the lack of any validated tool to inquire patients’ satisfaction. Indeed, investigators were forced to use non-validated questionnaires to evaluate functional outcomes.

Penetration ability (of those patients who had their implants remaining in situ) ranges from 85 to 93% and compares favourably to other implants in phalloplasty 23–77% [3, 11].

Recently, Verla et al. described their initial experience with ZSI 475 FtM PP in 39 trans-men and 7 cis-men after a median follow up of 12 months (SD 8–18) [15]. Infection (11%), mechanical failure (4.3%) and malposition (4.3%) and distal cylinder protrusion (2.2%) were causes for revisional surgery with explant in 20% of cases. The reported explant free implant survival rate was 83% at 6, 12 and 18 months respectively

That said, there is very limited data available and factors such as personal/relationship situation, psychological factors and phalloplasty dimensions may play a role.

In order to active the ZSI 475 FTM PP, ~60% of patients were fully satisfied with the pump function [14] in one study whilst 100% of patients in our small series felt that the device was easy to cycle.

Orgasm (ZSI PP) has been achieved in 60% of patients [14], this value may appear low compared to general PP implanted series but it is our opinion however, that other factors are involved here rather than the PP itself in trasngender patients.

Overall satisfaction rates have been reported with 93% of patients either satisfied/very satisfied with the ZSI device [14], comparable to 88% reporting full satisfaction for a mixed cohort of AMS and Coloplast implants [3].

Our own experience, between April 2019 and January 2020, of five patients, with median follow up of 12 months [IQR 8–17], demonstrates that all patients still had their original prosthesis in place. Median age at implantation was 32 [IQR 19–48] years and median time elapsed between phalloplasty and PP implantation was 12 months [IQR 8–20]. Median operative time was 95 min [80–120] and the distal silicon stopper needed to be modified in a single case to ensure an appropriate fit. No device infection occurred in our series and none required revision surgery. In one case, malposition led to a minor dorsal phallus curvature (<30°), which was managed conservatively; the patient was able to engage in penetrative sexual activity. PRO’s were evaluated through a non-validated satisfaction questionnaire (Table 2). Overall, all patients were able to cycle the device, 4/5 (80%) patients had engaged in penetrative sexual intercourse, all of whom had no pain during intercourse. 3/5 (60%) had achieved orgasm and 4/5 (80%) were fully satisfied with cosmetic and functional outcome of the total phallic reconstruction. Although these numbers and the duration of follow up limited, it compares with other published data.

**Table 2:** The non validated questionnaire to assess patient and partner satisfaction.

1-Do you have satisfactory sensation in the phallus?	Yes	No
2-Do you find easy to inflate the prosthesis?	Yes	No
3-Do you find easy to deflate the prosthesis?	Yes	No
4-Are you satisfied with the result?	Yes	No
5-Have you had penetrative sexual intercourse?	Yes	No
6-Do you manage to reach orgasm?	Yes	No
7-Is your partner satisfied with the result?	Yes	No

Thus whilst the phalloplasty surgical community has long awaited dedicated technologies and implants, the ZSI-475 FTM appears to claim some benefits. However, little can be solidly concluded at this early stage as we eagerly await further objective, well planned trials to assess both surgical complications, implant survival, and patient reported outcomes. May this be the first of a series, of exciting new developments for trans phalloplasty prosthetics, brought to fruitition together with well documented clinical and subjective outcome reporting.
